# Association of specific biotypes in patients with Parkinson disease and disease progression

**DOI:** 10.1212/WNL.0000000000010498

**Published:** 2020-09-15

**Authors:** Linbo Wang, Wei Cheng, Edmund T. Rolls, Fuli Dai, Weikang Gong, Jingnan Du, Wei Zhang, Shouyan Wang, Fengtao Liu, Jian Wang, Peter Brown, Jianfeng Feng

**Affiliations:** From the Institute of Science and Technology for Brain-inspired Intelligence (L.W., W.C., E.R., F.D, W.G., J. D., W.Z., S.W., J.F.), Fudan University; Key Laboratory of Computational Neuroscience and Brain-Inspired Intelligence (L.W., W.C., J. D., W.Z., S.W., J.F.) (Fudan University), Ministry of Education, Shanghai, China; Department of Computer Science (E.R., J.F.), University of Warwick, Coventry; Oxford Centre for Computational Neuroscience (E.R.), UK; Department of Neurology and National Clinical Research Center for Aging and Medicine (F.L., J.W.), Huashan Hospital, Fudan University, Shanghai, China; and Medical Research Council Brain Network Dynamics Unit (P.B.) and Nuffield Department of Clinical Neurosciences (P.B.), University of Oxford, UK.

## Abstract

**Objective:**

To identify biotypes in patients with newly diagnosed Parkinson disease (PD) and to test whether these biotypes could explain interindividual differences in longitudinal progression.

**Methods:**

In this longitudinal analysis, we use a data-driven approach clustering PD patients from the Parkinson's Progression Markers Initiative (n = 314, age 61.0 ± 9.5, years 34.1% female, 5 years of follow-up). Voxel-level neuroanatomic features were estimated with deformation-based morphometry (DBM) of T1-weighted MRI. Voxels with deformation values that were significantly correlated (*p* < 0.01) with clinical scores (Movement Disorder Society–sponsored revision of the Unified Parkinson’s Disease Rating Scale Parts I–III and total score, tremor score, and postural instability and gait difficulty score) at baseline were selected. Then, these neuroanatomic features were subjected to hierarchical cluster analysis. Changes in the longitudinal progression and neuroanatomic pattern were compared between different biotypes.

**Results:**

Two neuroanatomic biotypes were identified: biotype 1 (n = 114) with subcortical brain volumes smaller than heathy controls and biotype 2 (n = 200) with subcortical brain volumes larger than heathy controls. Biotype 1 had more severe motor impairment, autonomic dysfunction, and much worse REM sleep behavior disorder than biotype 2 at baseline. Although disease durations at the initial visit and follow-up were similar between biotypes, patients with PD with smaller subcortical brain volume had poorer prognosis, with more rapid decline in several clinical domains and in dopamine functional neuroimaging over an average of 5 years.

**Conclusion:**

Robust neuroanatomic biotypes exist in PD with distinct clinical and neuroanatomic patterns. These biotypes can be detected at diagnosis and predict the course of longitudinal progression, which should benefit trial design and evaluation.

Patients with Parkinson disease (PD) present heterogeneous motor and nonmotor clinical manifestations and have a variable prognosis.^1,2^ Although the diagnosis of PD is dependent on the presence of tremor, bradykinesia, and rigidity, some nonmotor phenomena—e.g., REM sleep behavior disorder (RBD), hyposmia, and depression—can precede motor deficits by several years. Conversely, as the disease progresses, nonmotor problems such as autonomic disturbances, sleep disorders, and cognitive impairment can dominate the clinical picture in some patients.^1^ Recent evidence suggests that PD may have several biotypes,^3–9^ but their identity and neurobiological basis remain poorly understood.^2^ Assuming that homogeneous groups of patients are more likely to share pathologic features, recognition of different subcategories of patients with PD may be key to better understanding underlying biological mechanisms, predicting disease profile and progression, and eventually designing more efficient personalized clinical trials.^2,3^

Subtypes of PD have previously been defined mainly according to clinical symptoms and demographic characteristics.^3–8^ However, cluster results are only as good as the data that underpin them, and the depths of phenotypic information used by these studies were variable, resulting in quite heterogeneous and controversial clusters.^2^ In addition, these clinical data-driven PD subtype classification systems may suffer from lack reproducibility.^10^ An alternative to subtyping patients with PD on the basis of co-occurring clinical symptoms is to identify neuroanatomic biotypes by clustering patients according to shared neuroanatomic signatures, which can objectively capture different aspects of patient characteristics. Studying brain neuroanatomic patterns of PD provides an opportunity to examine biological heterogeneity in vivo.^11^ Data-driven methods provide an unbiased approach to detect groups of patients with similar profiles across multiple neuroanatomic feature dimensions and thus may yield a more refined description of heterogeneity in PD. T1-weighted MRI is an especially suitable modality to describe brain anatomy with high resolution and to quantify regional brain volumes.^12^ Brain volume may mediate brain reserve, which promotes the resilience of large-scale brain networks and helps maintain normal function in the face of neurodegeneration.^13–16^ Previous studies have shown that subcortical volume loss reflects clinical measures of disease severity and is related to the development of cognitive impairment.^17–21^ These studies raise the intriguing possibility that T1-weighted MRI measures of brain volume could be leveraged to identify biotypes of PD. Critically, such PD biotypes defined by brain volume at diagnosis may predict disease progression, which may be advantageous in helping to determine prognosis and to identify subgroups for clinical trials. Cluster analysis in nondemented PD with limited sampling of patients showed different PD cortical thinning subtypes.^22^ These PD subtypes also showed different cortical thinning progression over time, but the difference between motor symptoms and the rates of disease progression of the different subtypes was not reported. Severity and rate of disease progression are an important issue in PD therapeutics, and identifying progression biotypes of PD at diagnosis with the use of neuroanatomic patterns may be one way to address heterogeneity in PD.

In this study, we used data-driven clustering approaches to identify neuroanatomic biotypes in patients with early PD in the Parkinson's Progression Markers Initiative (PPMI; ppmi-info.org) database^23^ according to the neuroanatomic pattern derived by deformation-based morphometry (DBM).^24^ DBM is based on nonlinear and intensity-based registration procedures that spatially normalize the entire brain to a standard template.^24^ DBM does not assume the distributions of gray matter or white matter and preserves the entirety of the MRI data. PD involves axonal degeneration and neuronal cell death, with the latter being indexed by gray matter atrophy, which is a relatively late event in the pathogenesis of PD.^25^ Moreover, neurodegeneration in PD initially preferentially affects subcortical regions through a purported disease-spreading process.^26^ A particularly strong aspect of the DBM method is that it enables the detection of subcortical neuroanatomic features,^27^ and previous studies have shown that DBM can detect morphologic tissue changes in patients with early-stage PD.^19^ Therefore, DBM is particularly suitable for PD biotype discovery compared to cortical thinning patterns and voxel-based morphometry. We hypothesize that if heterogeneity in clinical symptoms reflects true neuroanatomic biotypes of PD, then such neuroanatomic biotypes should be detectable in early disease and might predict the type of symptoms or disease progression that a patient will develop. The aims of our study were (1) to identify biotypes of PD with cluster analysis based on a baseline neuroimaging dataset; (2) to introduce a practical clinical typing method, which assigns individual patients to their biotype; and (3) to compare the behavioral assessments and rate of disease progression between different PD biotypes.

## Methods

### Standard protocol approvals, registrations, and patient consents

The study was approved by the institutional review board at each PPMI site. Written informed consent for research was obtained from all participants in the study.

### Overall design

**Figure 1:**
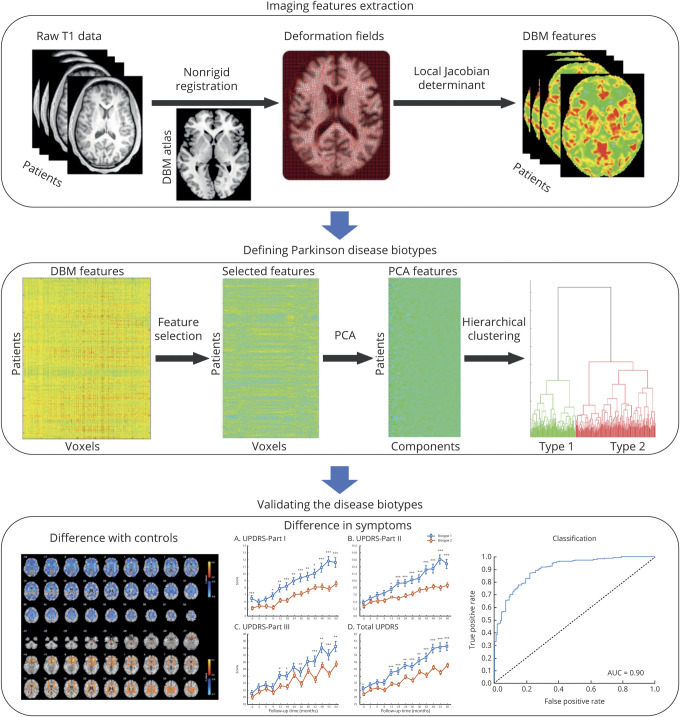
Schematic overview of the design of this study DBM = deformation-based morphometry; PCA = principal component analysis; UPDRS = Unified Parkinson’s Disease Rating Scale.

A flowchart of the analysis is shown in figure 1. We began by designing and implementing a preprocessing procedure to control for site- and age-related effects in a multisite dataset that comprised structural MRI scans for 457 participants (n = 314 patients with PD, n = 143 healthy controls). A graphic summary of the participants selection is shown in data available from Dryad (figure e-1, doi.org/10.5061/dryad.xsj3tx9bf). Patients and controls were matched for age and sex. DBM was used to detect the volume of each voxel compared to the template brain.^24^ Next, to select features for use in clustering, we used Spearman rank correlation analysis to identify a low-dimensional representation of neuroanatomic features that were associated with baseline clinical symptoms within patients with PD, including Movement Disorder Society–sponsored revision of the Unified Parkinson’s Disease Rating Scale (MDS-UPDRS) Parts I, II, III, tremor, and postural instability and gait disorder (PIGD) scores. To capture more neuroanatomic features related to PD, correlations were not corrected for multiple comparisons, but the dimensions of selected features were further reduced by principal components analysis. Then, hierarchical clustering was used to discover clusters of patients according to the principal components. Finally, to validate the clustering results, we investigated differences in follow-up clinical symptoms and neuroanatomic patterns between subgroups of patients, and we investigated the extent to which the analysis could reliably discriminate between different subgroups of patients using a pattern classification approach.

### Study setting and patients

The PPMI (ppmi-info.org) is a landmark observational, longitudinal database consisting of neuroimaging, biological tests, and clinical and behavioral assessments in >400 patients with de novo PD.^23^ All patients underwent dopamine transporter (DAT) imaging, and the diagnosis was confirmed by the DAT deficit. All clinical features were reassessed annually over 5 years so that markers of disease progression could be discerned. The neuroimaging data and extensive longitudinal clinical information provide an unprecedented opportunity to identify the neuroanatomic biotypes of PD and the longitudinal assessment of PD progression of different biotypes in patients with de novo PD. The clinical and behavioral assessments have been described extensively elsewhere.

Recruitment criteria included age >30 years, PD diagnosis within the last 2 years, baseline Hoehn and Yahr stage I to II, and no anticipated need for symptomatic treatment within 6 months of baseline.^23^ Clinical assessments were performed at baseline, at 3-months interval during the first year of participation, and then every 6 months thereafter (see data available from Dryad, table e-1, doi.org/10.5061/dryad.xsj3tx9bf). Data from the PPMI database were obtained in May 2018 in compliance with the PPMI Data Use Agreement.

### Baseline and clinical assessments

A comprehensive set of clinical assessments were evaluated in PPMI. We focused on clinical features that capture major PD symptoms, including both motor and nonmotor symptoms, in line with previous studies.^3,7,28,29^ Details of clinical assessments used in this study are presented in PPMI (ppmi-info.org/wp-content/uploads/2010/04/PPMI-General-Operations-Manual.pdf). Derived variable definitions and score calculations are available in the PPMI (in the Study_Docs). A list of abbreviations of clinical assessments is given in data available from Dryad (table e-2, doi.org/10.5061/dryad.xsj3tx9bf). Features captured include the following:

1. Demographics: age, sex, race, symptom duration, and education level.
2. Blood biomarkers: biochemical tests.
3. Motor: MDS-UPDRS Parts I through III,^30^ tremor/PIGD motor phenotype, PIGD, tremor subscale, and Schwab-England activities of daily living scores.
4. Cognitive testing: cognitive function (age/education adjusted Montreal Cognitive Assessment total score)^31^ and neuropsychological variables, including visuospatial and visuoperceptual functions (Benton Judgment of Line Orientation),^32^ cognition performance (Symbol Digit Modalities Test),^33^ verbal learning and memory (Hopkins Verbal Learning Test for total recall, delayed recall, retention, and recognition-discrimination),^34^ semantic memory (Semantic Verbal Fluency test),^34^ and working memory capacity (letter-number sequencing).^35^
5. Autonomic testing: autonomic dysfunction (Scales for Outcomes in Parkinson's Disease–Autonomic total score and its subscores: cardiovascular, constipation, orofacial, thermoregulatory, sexual, pupillomotor, and urinary).^36^
6. Sleep disorders: RBD (RBD Screening Questionnaire score),^37^ average sleep propensity in daily life (Epworth Sleepiness Scale score).^38^
7. Neurobehavior: depression (Geriatric Depression Scale score),^39^ trait and state anxiety (State-Trait Anxiety Inventory score),^29^ and impulse control disorders and related disorders (Questionnaire for Impulsive-Compulsive Disorders in Parkinson's Disease score).^40^
8. Olfactory testing: impaired olfaction (age/sex adjusted University of Pennsylvania Smell Identification Test score).^41^
9. Physical activity: Physical Activity Scale of the Elderly.^42^ Three activity categories were assessed: leisure, household chores, and work/volunteering.

### CSF and SPECT biomarkers

A lumbar puncture was conducted for all participants for the collection of CSF. β-Amyloid_1–42,_ phosphorylated tau, and total tau protein were measured by INNO-BIA AlzBio3 immunoassay (Innogenetics Inc, Ghent, Belgium), and α-synuclein concentration was measured by ELISA. SPECT with the DAT tracer ^123^I-ioflupane was acquired at baseline and follow-up visits.^23^

### Imaging processing

T1-weighted MRI scan acquisition parameters are detailed elsewhere (ppmi-info.org/wp-content/uploads/2017/06/PPMI-MRI-Operations-Manual-V7.pdf).

The T1-weighted MRI images were preprocessed with the Computational Anatomy Toolbox (CAT 12) (dbm.neuro.uni-jena.de/cat12/), which is an extension of SPM12 to provide computational anatomy. All these images were corrected for bias, noise, and intensity and linearly and then nonlinearly registered to the Montreal Neurological Institute 152-2009c template. Then the DBM (i.e., the determinant of the jacobian transformation matrix) maps were calculated to estimate the local volume in each voxel (DBM values). Raw images of lower quality (CAT image quality rating <75%) were excluded. The rest images were further visually checked. Finally, the obtained preprocessed volume-based DBM data from 314 patients with PD and 145 healthy controls were smoothed with an 8-mm full width at half-maximum.

For volumetric analysis, FreeSurfer version 5.3 was used to derive measures of the volume of subcortical nuclei. This is a well-documented and freely available software.^12^

### Voxel-level association study and clustering

We reasoned that a low-dimensional representation of a subset of neuroanatomic features would best characterize biologically meaningful PD biotypes, similar to the atrophy subtypes detected in prodromal Alzheimer disease.^11^ Therefore, to select a set of neuroanatomic features for use in clustering, we used Spearman rank correlation analysis to identify features that were significantly correlated (*p* < 0.01) with clinical scores (baseline value): the MDS-UPDRS Parts I to III, UPDRS total, tremor, and PIGD scores. Confounding factors such as age, sex, years of education, race (categorized as white or other), and site effect were regressed out before feature selection.

To further exclude undesired background noise, principal component analysis was used to extract a lower-dimensional component space of the selected features (79 principal components were used capturing 90% of the variance). Then we used hierarchical clustering to assign participants to nested subgroups with similar pattern. We calculated a similarity matrix describing the correlation distance between every pair of participants, and then we used the Ward minimum variance method to iteratively link pairs of participants in closest proximity, forming progressively larger clusters in a hierarchical tree. Calinski-Harabasz criterion values were used to estimate the optimal number of clusters, and the result suggested 2 clusters as the best choice (see data available from Dryad, figure e-2, doi.org/10.5061/dryad.xsj3tx9bf). Furthermore, to validate the clustering, we also clustered data using k-means clustering. The Cohen κ agreement rate between hierarchical clustering and k-means clustering was 0.68, which is in the substantial range, suggesting that the patterns identified by the 2 different clustering methods were similar (see data available from Dryad, figure e-3).

### Classification

To further test the clinical relevance of the identified neuroanatomic features as diagnostic features of biotypes, we applied a support vector machine to test how well this could discriminate these 2 biotypes, that is, classify individuals into 1 of these 2 subgroups. A 10-fold cross-validation strategy was used to estimate its accuracy, sensitivity, and specificity. The details of classification are depicted in data available from Dryad (figure e-4, doi.org/10.5061/dryad.xsj3tx9bf).

### Statistical analyses

#### Demographics and clinical variables

The *t* test was used to determine the statistical significance of continuous demographic and clinical variables after the removal of confounding variables: age, sex, years of education, race (categorized as white or other), and site effect. The χ^2^ test was used to test the significance of categorical demographic variables and phenotype variables. Statistical significance was established at *p* < 0.05 (false discovery rate [FDR] correction), and the values were reported as mean (SD) for each demographic and clinical variable. Missing data were not included in all analysis.

#### Linear mixed model fitting for disease progression rates

We estimated rates of progression for each patient with 5 years of follow-up. Linear mixed models were used to evaluate baseline and disease progression rates over time in patients classified in the subtypes using the lme4 package in R.^43^ Sex, age, sites, race, time from baseline (months), biotype, and their interaction were included as fixed effects. Participant intercepts and slopes (rates of progression) were modeled as random effects.

### Data availability

All deidentified clinical and imaging data are available on the PPMI website (ppmi-info.org) and from the corresponding author on reasonable request.

## Results

### Baseline dataset characteristics

A total of 314 early patients with PD were included in this study consisting of 207 (65.9%) men and 107 (34.1%) women. On average, these patients with PD were 61.0 ± 9.5 years of age with a disease duration (date of enrollment minus the date of diagnosis) of 6.9 ± 6.8 months at baseline. The mean MDS-UPDRS Parts I through III scores were 5.6 ± 4.0, 5.9 ± 4.2, and 20.7 ± 8.7. Clinical, biological, and cognitive characteristics of early patients with PD and matched healthy controls are given in table 1.

**Table 1:**
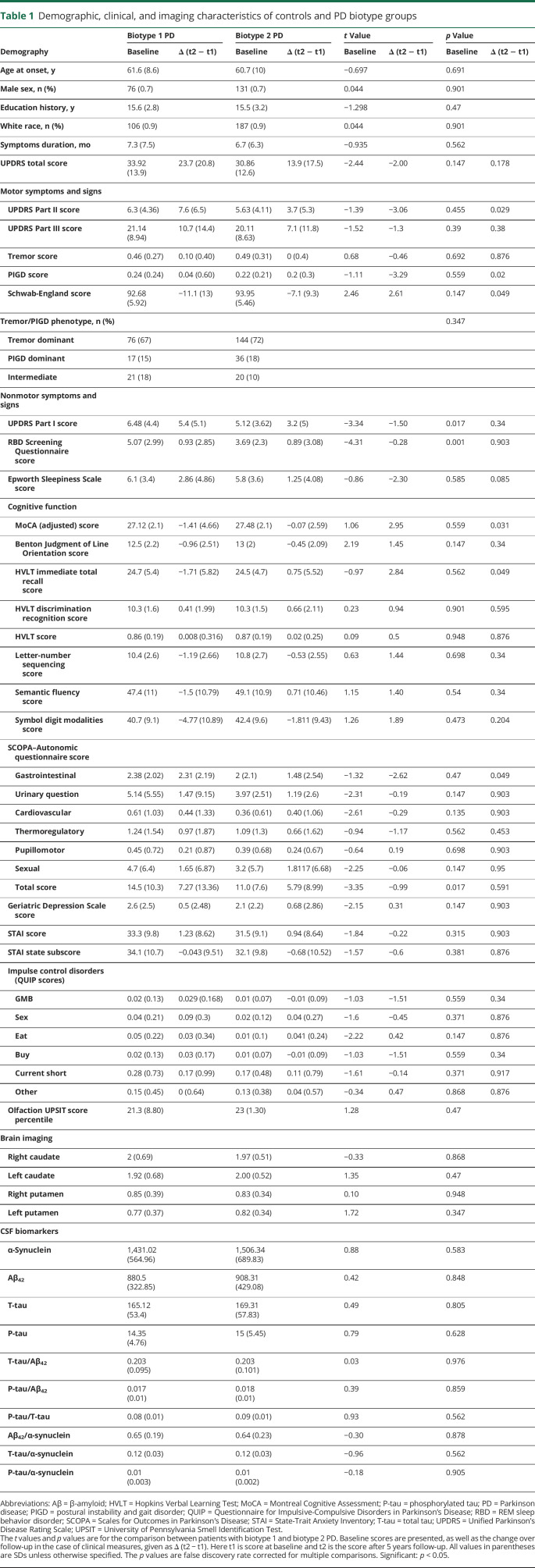
Demographic, clinical, and imaging characteristics of controls and PD biotype groups

### Neuroanatomic features associated with the symptoms of PD

**Figure 2:**
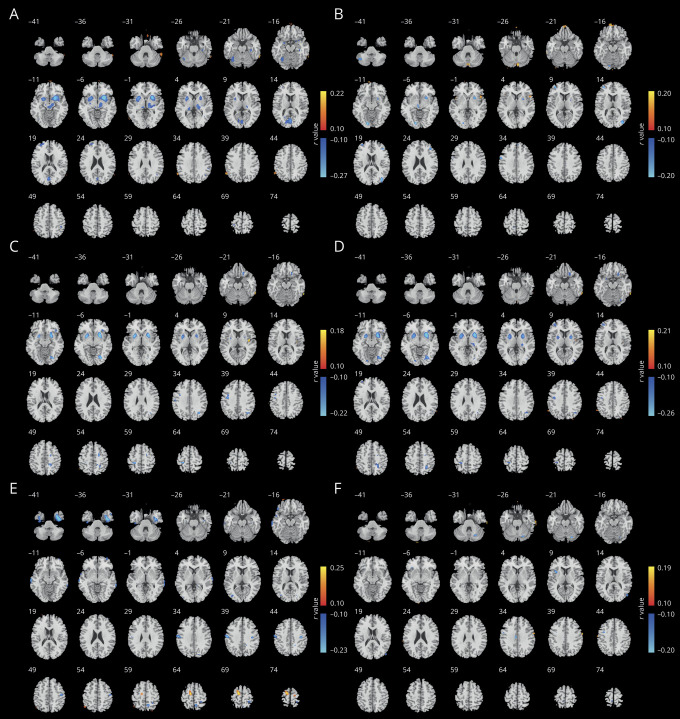
Correlation between DBM values and symptom scores Warm color indicates a positive correlation between symptom scores and deformation-based morphometry (DBM) values; cold color indicates a negative correlation between symptom scores and DBM values. (A) Correlation between DBM values and Movement Disorder Society–sponsored revision of the Unified Parkinson’s Disease Rating Scale (MDS-UPDRS) Part I score. (B) Correlation between DBM values and MDS-UPDRS Part II score. (C) Correlation between DBM values and MDS-UPDRS III score. (D) Correlation between DBM values and MDS-UPDRS total score. (E) Correlation between DBM values and tremor score. (F) Correlation between DBM values and postural instability and gait disorder (PIGD) score. In total, 8,855, 2,826, 4,201, 6,064, 6,994, and 1,380 deformation values correlated with UPDRS Part I, UPDRS Part II, UPDRS Part III, total UPDRS, PIGD, and tremor scores, respectively (*p* < 0.01, uncorrected).

Figure 2 shows the correlation analysis between deformation values and baseline clinical scores. We found that a number of neuroanatomic features correlated with UPDRS scores, including areas spanning the caudate, putamen, thalamus, hippocampus, supplementary motor area, and orbital frontal gyrus (figure 2). These areas are consistent with previous findings that subcortical volume loss correlates with motor symptom severity.^18^ This empirical, data-driven approach to feature selection identified 23,213 voxel DBM values that were correlated with at least 1 baseline clinical score (figure 2).

### Brain neuroanatomic patterns define 2 PD biotypes

**Figure 3:**
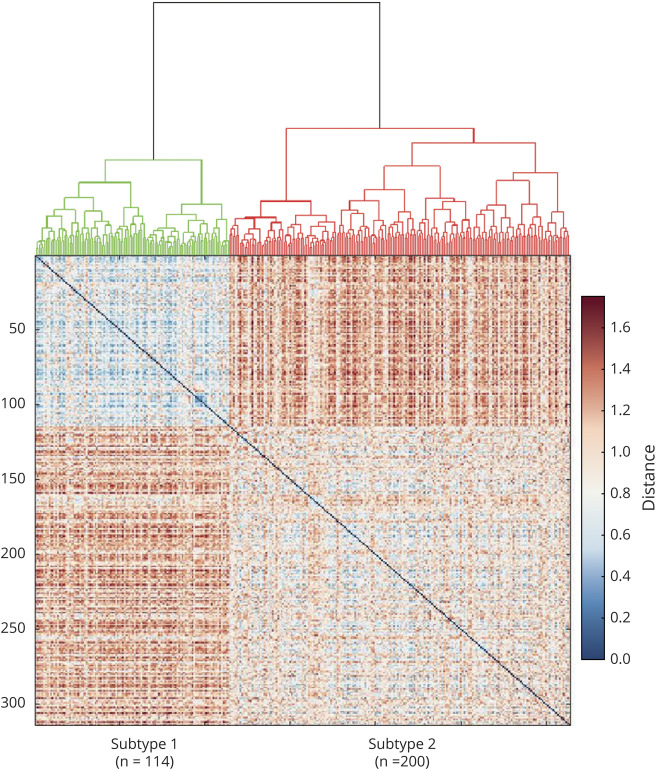
Dendrogram of the final hierarchical cluster solution of patients with PD in the PPMI patients Matrix under the dendrogram shows the distance of the neuroanatomic patterns between different patients with Parkinson disease (PD). Each element (i row and j column) in the matrix indicates the difference of the neuroanatomic patterns between the i participant and j participant. PPMI = Parkinson's Progression Markers Initiative.

We then tested whether these neuroanatomic feature sets tended to cluster in patient subgroups. As illustrated in figure 3 and data available from Dryad (figure e-3, doi.org/10.5061/dryad.xsj3tx9bf), the cluster analysis revealed 2 distinct clusters of patients with PD, with similar disease duration (*p* = 0.35, table 1). These 2 clusters comprised 36.31% (114 patients) and 63.69% (200 patients) of the 314 patients with PD. Table 1 shows the demographic characteristics of the 2 biotype groups. There was no significant difference in age, sex, education, symptom duration, or ratio of PIGD- and tremor-dominant patients between the 2 biotype groups (table 1 and data available from Dryad, table e-3).

### Neuroanatomic pattern of the 2 biotypes

**Figure 4:**
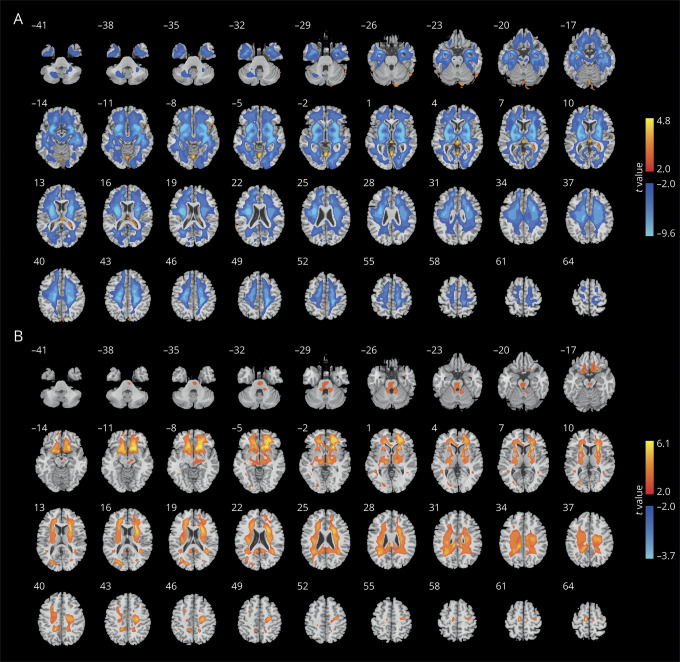
Different neuroanatomic patterns in the 2 PD biotypes compared with healthy controls Warm color indicates higher deformation-based morphometry (DBM) values in patients with Parkinson disease (PD); cold color indicates lower DBM values (*p* < 0.005, false discovery rate correction). (A) The *t* values of comparison of DBM values between biotype 1 and controls. (B) The *t* values of comparison of DBM values between biotype 2 and controls.

To illustrate the neuroanatomic pattern in the different PD biotypes, the DBM values of the 2 biotypes were compared with those of controls. Compared to normal controls, individuals with both biotypes showed significant differences in subcortical regions. Figure 4 shows the comparison between each biotype and healthy controls (*p* < 0.005, FDR correction).

Those with biotype 1 had pronounced differences compared to normal controls in almost the whole brain, indicating more severe atrophy in brain areas in early PD. Those with biotype 2 had predominant differences in the subcortical regions. In addition, we found other distinct patterns that differentiated the 2 biotypes. For example, compared to controls, DBM values in the putamen, caudate, pallidum, lingual gyrus, temporal cortex, insula, amygdala, hippocampus, and orbital frontal cortex, which regulate motor-related, cognitive, and emotion-related behavior, were significantly lower in patients with biotype 1, which were characterized in part by increased motor and nonmotor symptom scores. In contrast, patients with biotype 2 had significantly higher DBM values in the brainstem, putamen, caudate, occipital lobe, lingual gyrus, olfactory, posterior cingulate cortex, and white matter areas compared to controls. Additional volumetric analyses with FreeSurfer showed that patients with biotype 1 had significantly lower subcortical volumes within the thalamus, caudate, putamen, pallidum, accumbens, amygdala, and hippocampus (*p* < 0.05, FDR correction) compared to patients with biotype 2 (see data available from Dryad, table e-4, doi.org/10.5061/dryad.xsj3tx9bf).

### Neuroanatomic patterns for diagnosing PD biotypes

We reasoned that clustering could be used for training the classifiers for the diagnosis of PD biotypes solely on the basis of structural MRI measures. To this end, we trained classifiers for predicting the PD biotype in individual patients. Ten-fold cross-validation was used to assess performance and significance. Support vector machine classifiers (using gaussian kernel functions) yielded overall accuracy rates of 84.1% (sensitivity 0.71, specificity 0.89, area under the curve 0.90, data available from Dryad, figure e-6, doi.org/10.5061/dryad.xsj3tx9bf) for the clusters characterized above.

### Baseline differences in symptoms between biotypes

Table 1 shows that at baseline patients with biotype 1 had worse mentation, behavior, and mood (higher MDS-UPDRS Part I score) and very much worse RBD Screening Questionnaire score than those with biotype 2. There was also evidence of more severe autonomic function in biotype 1 (Scales for Outcomes in Parkinson's Disease total score) (table 1).

### Disease progression in the PD biotypes

**Figure 5:**
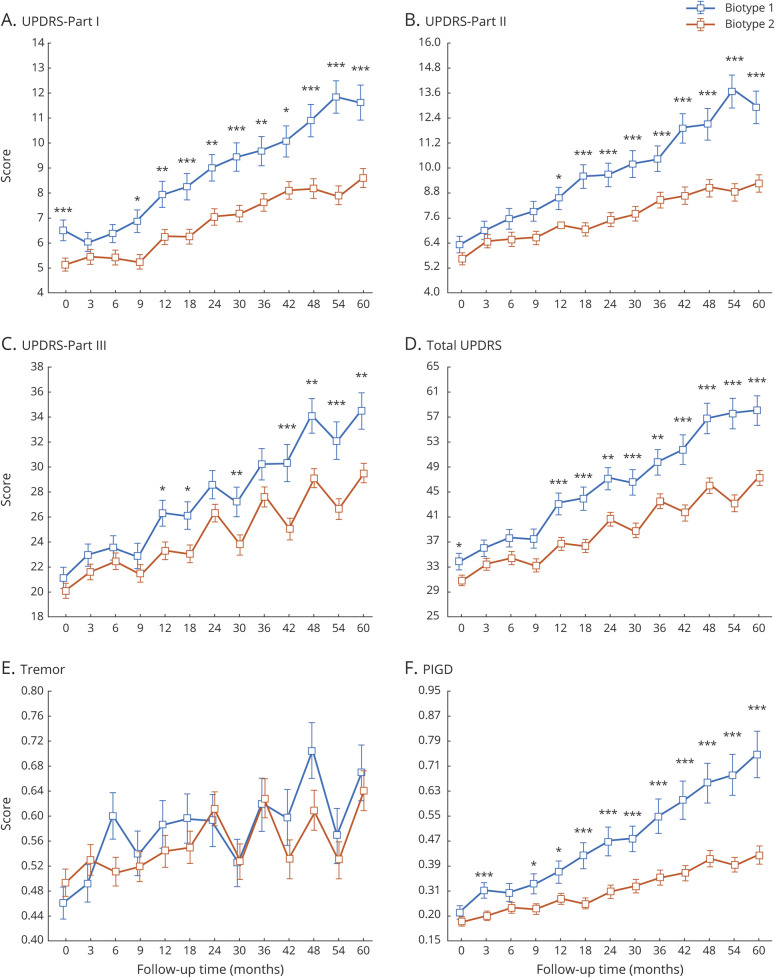
Longitudinal changes in outcomes of interest in different biotypes of PD among the PPMI patients with 5 years of follow-up Asterisks indicate the statistical significance of the comparison between 2 biotypes in the clinical variables at the time of different follow up (A–F; *p < 0.05, **p < 0.01, ***p < 0.005, uncorrected for multiple comparisons). The Parkinson's Progression Markers Initiative (PPMI) data contain 1 baseline set of data and 12 follow-up sets of data over 5 years. PD = Parkinson disease; PIGD = postural instability and gait disorder; UPDRS = Unified Parkinson’s Disease Rating Scale.

The PPMI patients were followed up for 5 years. The sample size of the progression analysis is shown in data available from Dryad (table e-1, doi.org/10.5061/dryad.xsj3tx9bf). Results from a linear mixed model showed that the patients with biotype 1 had significantly greater progression in all MDS-UPDRS scores, with the exception of the tremor score (figure 5 and table 2). In addition, those with biotype 1 tended to develop more severe cognitive impairment (Hopkins Verbal Learning Test immediate total recall, letter-number sequencing, and Symbol Digit Modalities) (table 2 and data available from Dryad, figure e- 8). More rapid progression could also be seen in the patients with biotype 1 in activities of daily living (Schwab-England activities of daily living) (table 2 and data available from Dryad, figure e-7).

**Table 2:**
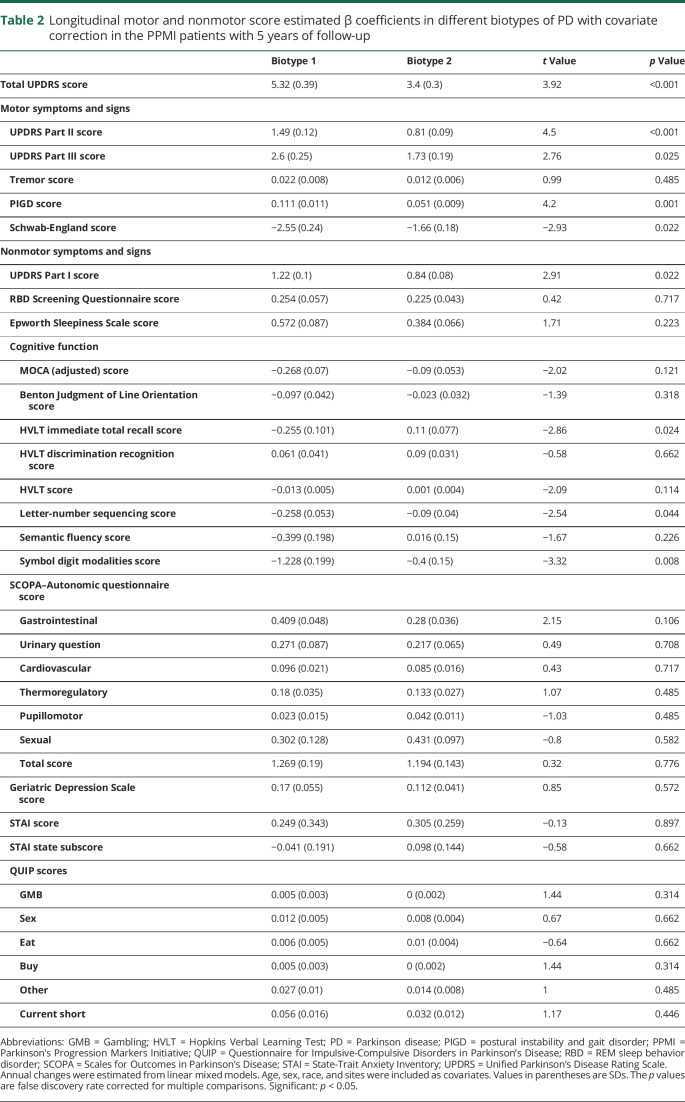
Longitudinal motor and nonmotor score estimated β coefficients in different biotypes of PD with covariate correction in the PPMI patients with 5 years of follow-up

On dopaminergic SPECT scanning, patients with biotype 1 had worse denervation of both left caudate and left putamen after an average of 4 years of follow-up. The right caudate and right putamen showed no significant difference in denervation between the 2 biotypes (see data available from Dryad, figure e-13, doi.org/10.5061/dryad.xsj3tx9bf).

To summarize, patients with biotype 1 had higher baseline MDS-UPDRS Part I score, worse baseline sleep problems and autonomic dysfunction, and faster progression of most motor symptoms, cognitive impairment, and activities of daily living compared to patients with biotype 2 (all *p* < 0.05, FDR correction) (figure 5 and tables 1 and 2).

## Discussion

In this study, we identified 2 neuroanatomic biotypes in patients with PD with otherwise similar demographics using an unbiased data-driven clustering approach applied to the PPMI cohort. Neuroanatomic biotypes differed in symptomatology even at presentation before treatment and thereafter progressed at different rates. The most striking baseline difference was the much higher RBD Screening Questionnaire score in biotype 1.

Our results provide support for 2 different neuroanatomic phenotypes within patients with PD. Compared to healthy controls, the neuroanatomic differences were more widespread in patients with biotype 1, spanning across almost all of the brain. Compared to patients with biotype 1, patients with biotype 2 had less widespread differences at baseline.

Patients with biotype 2 had larger subcortical volume (higher DBM values) than controls on average, suggesting they may contain more cells (including dopaminergic cells) and synapses, increasing the ability to support maintenance of function despite declines in brain volume. Accordingly, patients with biotype 2 had a slower disease progression rate.^3^ In contrast, patients with biotype 1 had less brain reserve and did not compensate as well as those with biotype 1 as PD progressed over time, resulting in a faster disease progression rate. Indeed, there was evidence that this was already the case at the time of presentation, given the worse symptom severity in several domains in biotype 1 than in biotype 2. In summary, we hypothesize that the different rates of symptom progression relate to different brain reserves.^13,16^

Brain reserve describes the differences in brain volume and structure that may support maintenance of function despite pathology.^13,44^ Gross or regional brain volume reflects the quantity of neurons, neuronal integrity, and synaptic densities, which determine the ability of the brain to engage in compensatory activity.^13,44^ Prior works have highlighted a link between brain volume and markers of functional reserve in patients with PD and other neurodegenerative diseases.^14–16^ For example, there is a relationship between brain gray matter volume and the magnitude of network-level integration.^16^ Basal forebrain volume can predict future psychosis in early PD, and higher cholinergic nucleus 4 gray matter density is associated with a lower risk of reporting psychotic symptoms.^17^ PD with cognitive impairment shows lower gray matter volume in the nucleus basalis of Meynert.^20^ Compared to tremor-dominant patients, patients with PIGD had lower gray matter volumes in the globus pallidus and amygdala and have worse prognosis with a more rapid decline.^45,46^ In line with these observations, our results suggest that a larger subcortical volume helps limit the negative impact of PD pathology during disease progression, as represented by brain atrophy. Physical exercise has been shown to increase brain volume in older adults.^47^ Therefore, interventions that increase physical activity before or in the early course of PD may contribute to brain reserve and help slow the rates of disease progression.

At baseline, patients with biotype 1 had evidence of worse behavioral, autonomic, and motor impairment and, above all, of worse RBD symptomatology. These findings are consistent with past observations showing that motor dysfunction is associated with cognitive decline, autonomic dysfunction, and RBD.^28,48^ PD with RBD is also associated with faster motor progression and a higher risk of cognitive decline.^28^ In line with these studies, we found that patients with biotype 1 had much worse RBD symptomatology and more cognitive decline. RBD may be a useful marker for early subtyping of PD at baseline.^28^ Patients with biotype 1 had significantly higher scores in several motor disease symptoms after only 1 year of follow-up, but there was no difference in the progression of tremor between the 2 biotypes. This may reflect that rest tremor may be more closely related to degeneration of nondopaminergic, rather than dopaminergic, systems.^49^ In most cases, there is a substantial asymmetry of clinical symptoms from disease onset, and patients with unilateral disease showed a significant difference in striatal uptake between the ipsilateral and contralateral sides in both the caudate and putamen nuclei.^49^ Differences in longitudinal denervation between the left and right caudate and putamen between the 2 biotypes (see data available from Dryad, figure e-12, doi.org/10.5061/dryad.xsj3tx9bf) may reflect different disease severity. Consistent with previous findings that CSF biomarkers are not useful biomarkers of PD progression,^50^ our results did not show significant differences in CSF biomarker levels between the 2 biotypes at baseline (table 1).

While the 2 identified neuroanatomic biotypes showed group differences in terms of symptom severity and longitudinal progression, there is overlap in the neuroanatomic features at the individual level between the 2 biotypes. It is plausible that some patients were a combination of >1 biotype, and this would not be captured by our approach of discretizing biotypes. Future research should further investigate more refined biotype definitions based on continuous membership probability values through longitudinal studies in larger cohorts. Therefore, we regard the 2 biotypes identified here as just an initial solution to the problem of diagnostic heterogeneity in a subtyping process that relies primarily on neuroanatomic features correlated with clinical scores. It is likely that cohort limitations (the PPMI patients on the whole have a higher level of education, are younger, and have less baseline disability than the general PD population^23^), the size of our cluster-discovery dataset, and the subjectivity of clinical-symptom assessments were also limiting factors. For these reasons, a novel cohort with longitudinal clinical data will be useful for validating the present findings.

We show that neuroanatomic biotypes can be defined that robustly predict different rates of progression, suggesting that these reflect true biotypes of PD. Given that PPMI recruited early patients from multiple sites, our results should still be mostly generalizable to early PD in real clinical practice where the findings can be used to inform estimates of prognosis. These results might also have implications for clinical trial design in early PD in the future. The existence of neuroanatomic biotypes that show specific trajectories of clinical score decline may require biotype-specific outcome measures tailored to the expected rate of decline in different domains.

We have robustly identified 2 different neuroanatomic biotypes among patients with early PD using a data-driven clustering approach. These biotypes showed distinct neuroanatomic patterns, symptoms, and rates of progression. Recognition of this heterogeneity is an important step toward precision medicine for PD.

## Glossary

DAT: dopamine transporter
DBM: deformation-based morphometry
FDR: false discovery rate
MDS-UPDRS: Movement Disorder Society–sponsored revision of the Unified Parkinson’s Disease Rating Scale
PD: Parkinson disease
PIGD: postural instability and gait disorder
PPMI: Parkinson's Progression Markers Initiative
RBD: REM sleep behavior disorder
